# The impact of perceived negative supervisor gossip on employee emotional exhaustion: a moderated mediation model of impression management and task interdependence

**DOI:** 10.3389/fpubh.2025.1575259

**Published:** 2025-04-02

**Authors:** Sheng Cheng, Yumei Wang, Chien-Chih Kuo

**Affiliations:** ^1^School of Business, Suzhou University of Science and Technology, Suzhou, China; ^2^Department of Psychology, National Chengchi University, Taipei, Taiwan

**Keywords:** negative supervisor gossip, impression management, emotional exhaustion, task interdependence, moderated mediation model

## Abstract

Negative gossip is a common behavior in the workplace. However, little research focuses on the employee perception of negative supervisor gossip in workplace. This study proposes that employee perceived negative supervisor gossip is a stressor that may lead employees to try coping using impression management tactics but ultimately to experience emotional exhaustion. Data were collected from 406 full-time Chinese employees and assessed with a time-lagged design. The results show that employee perceived negative supervisor gossip has a significant positive relationship with impression management, and impression management is positively correlated to emotional exhaustion. Furthermore, impression management mediates the relationship between perceived negative supervisor gossip and emotional exhaustion. Finally, task interdependence moderates the direct and indirect effects among perceived negative supervisor gossip, impression management, and emotional exhaustion. This study also discusses the theoretical and practical implications for managers. Specifically, supervisors should minimize the frequency of negative gossip behavior, encourage self-actualization among employees, and provide employees with more collaborative tasks.

## Introduction

Employee emotional exhaustion is a critical concern in organizational behavior (1, 2). Studies have shown that effective information exchanges in the workplace have a significant impact on employee emotional exhaustion (3–5). However, prior research has primarily focused on the formal communications in organizational behavior to mitigate employee emotional exhaustion (6–8), overlooking the impact of informal communication, such as negative workplace gossip (9–11).

Prior studies have demonstrated negative workplace gossip linked to increased work stress (12), employee cynicism (13), and diminished employee mental health and subjective well-being (14, 15). Although prior research has not pointed out a relationship between negative workplace gossip and emotional exhaustion, the effects of negative workplace gossip on employee work attitudes and psychological well-being suggest that it may have an indirect effect on emotional exhaustion. Therefore, the present study suggests that clarifying the relationship between negative workplace gossip and emotional exhaustion is an invaluable contribution to management practices.

Moreover, this study focuses on perceive negative workplace gossip from supervisors rather than peers. Prior studies more emphasized on coworker negative workplace gossip (10, 13, 15), ignoring the supervisor-subordinate communication may also significantly affect employee attitudes and performance (16, 17). Specifically, the present study suggests that negative supervisor gossip serves as criticism or unfavorable discussions about employees, which may act as a stressor, increasing anxiety and fear of being the next target of supervisor’s negative gossip (18). Therefore, when employees perceiving such gossip may experience heightened stress, leading to emotional exhaustion.

Based on the above discussion, this study intends to employ three stage (alarm, resistance, and exhaustion) of general adaptation syndrome theory (19) to explain the relationship and underlying mechanism between perceived negative supervisor gossip and employee emotional exhaustion. The general adaptation syndrome theory was initially developed to study the physiological effects of stress. However, an increasing number of studies have since applied it to explain individuals’ psychological stress (20, 21), especially in workplace context. Therefore, this study proposed that, when employee perceive negative gossip from supervisor, they may recognize it as psychological stressors, triggering the alarm stage and experiencing heightened anxiety and uncertainty. Then, employee may adopt strategies to try to cope with the stressor, which means they enter the resistance. In the resistance stage, they may engage in impression management (22) tactics to enhance their public appearance and avoid to become the next target of supervisor negative gossip. Finally, while impression management can temporarily alleviate stress, it consumes cognitive and emotional resources (23), potentially leading to exhaustion (24).

Furthermore, this study considers the moderating role of task interdependence refers to the degree to which tasks or job responsibilities in an organization are interconnected or dependent upon other employees (25). The present study proposes that employees with high interdependence may mitigate the stressful impact of gossip by diffusing perceptions of personal responsibility, resulting in less impression management strategies. In contrast, employees with low interdependence heightens individually stress due to their supervisors assign greater personal responsibility on them, leading them to exhibit more impression management behaviors.

**Figure 1:**
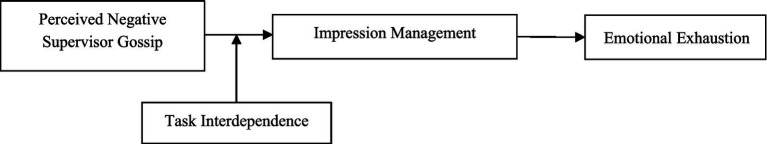
Proposed conceptual scheme.

In summary, the conceptual model of our study is presented in Figure 1. This study based on General Adaptation Syndrome as our theoretical framework, this study positions supervisor negative gossip as a salient workplace stressor that triggers employees’ stress responses of impression management, leading to emotional exhaustion. This study makes several contributions to the literature and to organizational behavior practices. First, we identify the relationship between perceived negative supervisor gossip and impression management, this contributes to the organizational behavior and stress literature by highlighting informal communication as a potential stressor that can lead to employee emotional exhaustion.

Second, our research expands the understanding of perceived negative supervisor gossip by investigating its impact on emotional exhaustion through impression management by applying general adaptation syndrome theory. This finding extends the academic knowledge and emphasize the how impression management serves as the underlying mechanism between perceived negative supervisor gossip and employee emotional exhaustion.

Third, the present study identifies the task interdependence as a boundary condition. This insight offers practical implications for managing informal communication and mitigating emotional exhaustion in the workplace.

## Theory and hypotheses

### Perceived negative supervisor gossip

Workplace gossip is a common informal communication behavior (11, 15) and is characterized by casually discussing and evaluating an absent individual’s attitude, behavior, or work performance (9, 10, 13). The research has divided workplace gossip behaviors into two types: positive and negative (13, 15, 26). Positive gossip refers to informal discussing an individual’s normative behaviors or positive reputation, such as an outstanding job performance, a job promotion, or the overcoming of challenges. In contrast, negative gossip refers to informal discussing an individual’s norm violations or negative reputation, such as poor performance, inadequate skills, or sloppy work (13, 15, 27). In recent years, researchers have shifted their focus on workplace gossip to explore employees’ reactions from an organizational leadership perspective (18, 28). Therefore, inspired by previous research, this study explores the impact of perceived negative supervisor gossip on employees.

Supervisors act as “meaning makers” in the workplace (18), so their behaviors are likely to influence employees’ perceptions of their own behaviors, attitudes, and performance through supervisors’ evaluations (29, 30). When supervisors engage in negative gossip about their employees, it is defined as using casual conversations to convey unfavorable evaluations of employees’ disappointing behaviors, attitudes, or job performance (27). Kuo et al. (18) and Xie et al. (31) suggested that employees perceive such negative supervisor gossip not as a direct experience of gossip themselves, but rather as a stressful situation implying that they might be the next target of such evaluations when they may not reach their supervisors’ standards and work requirements. This perception triggers a sense of anxiety and fear among employees, who worry about becoming the subject of future negative gossip. Furthermore, individuals who are exposed to such an environment of negative gossip in the workplace may suffer from psychological distress (14, 15) and emotional exhaustion (32).

Consequently, we propose that employees’ perception of supervisors engaging in negative gossip about subordinates as a potential threat to their reputation and standing at work are prompted to enter the alarm stage of stress (19). This stress response drives them to make fight-or-flight decisions in response to the perceived stressor of potential negative evaluations from their supervisors.

### The relationship between perceived negative supervisor gossip and impression management

General adaptation syndrome theory can also explain how employees respond to the stressor of perceived negative supervisor gossip. After entering the alarm stage due to the potential reputational harm and social threat by supervisor negative gossip (18, 31), employees may trigger a heightened stress response (33). Thus, they enter the resistance stage (19).

In the resistance stage, individuals engage in coping mechanisms to manage this stressor. One primary coping strategy is impression management (22, 34), which involves deliberate efforts to shape how others perceive them (22, 34). Such behavior is intended to create or uphold the image individuals wish to portray to others (23, 26, 35). Jones and Pittman (36) noted that individuals engage in impression management through the following five behavioral tactics: (1) self-promotion, in which individuals highlight their positive qualities, achievements, and capabilities to create a favorable public appearance; (2) ingratiation, in which individuals build positive social relationships by pleasing and gaining the favor of others (e.g., compliments and flattery); (3) exemplification, in which individuals simulate busyness, employing superficial tactics to highlight their moral values, sense of responsibility, and self-discipline to improve their public appearance; (4) intimidation, in which individuals use displays of authority to shape or maintain their influential and authoritative public appearance; and (5) supplication, in which individuals feign helplessness, fragility, or a need for assistance to elicit sympathy and support from others.

Negative supervisor gossip not only suggests unfavorable evaluations, but also indicates the potential to jeopardize their public appearance (33). Therefore, employees may seek to counteract by strategically managing their public image (23, 36). Research indicates that individuals use impression management to repair social perception and counter negative judgments, particularly when faced with reputational threats (23, 35, 37).

Furthermore, from self-regulation perspective, employees who perceive themselves as potential targets of negative gossip by their supervisors enter a state of heightened awareness and self-protection. In doing so, they seek to emphasize their strengths, achievements, and positive attributes while concealing any perceived weaknesses or vulnerabilities. This strategic behavior is aimed at building or preserving a favorable public image, as they attempt to mitigate the potential negative consequences of being the subject of supervisors’ casual chatter. Given that impression management serves as a protective mechanism against unfavorable evaluations, it becomes a logical coping response to perceived supervisor gossip.

Thus, based on in general adaptation syndrome theory and self-regulation mechanisms, this study proposes the following hypothesis:

*Hypothesis 1*: Perceived negative supervisor gossip is positively correlated with employee impression management.

### The relationship between impression management and emotional exhaustion

According to general adaptation syndrome theory (19), this study posits that engaging in impression management can lead employee to exhaustion stage due to the depletion of personal resources. To be specific, individuals respond to stressors by mobilizing resources in the alarm stage, sustain efforts in the resistance stage, and eventually experience exhaustion when resources are depleted.

Impression management is an effortful and strategic behavior requiring sustained self-regulation (23), which, over time, can drain cognitive and emotional resources of employees (34). Specifically, Individuals must heighten their sensitivity to social cues and remain vigilant in adjusting their behaviors to consistently achieve a positive public appearance that fit social demands (38). Moreover, individuals must exert additional effort in terms of self-control (26) and cognitive resources to ensure that others do not perceive their instrumental motives (39, 40), adding to the cognitive and emotional load, which can progressively lead to, emotional exhaustion.

Additionally, Bolino et al. (41) further indicated that employees typically aspire to be perceived as diligent and committed at work may cause their psychological strain. For example, an employee pretending dedication may stay late, act busy during slow times, arrive early, or even work evenings and weekends to show their devotion but without any affective commitment (42). Therefore, employees who continually present a fake public appearance of dedication to work and positive social relationships that contradict their true selves can deplete their own psychological resources and energy (36, 43, 44). When employees’ personal resources in the workplace are continually depleted without resource gain, the employees may experience emotional exhaustion (1, 6, 44–46). Thus, this study proposes the following hypothesis:

*Hypothesis 2*: Employee impression management is positively correlated with emotional exhaustion.

### The mediating role of impression management

This study uses general adaptation syndrome theory (19) to explain the effect of perceived negative supervisor gossip on emotional exhaustion. We propose that perceived negative supervisor gossip, as a workplace stressor (11, 15), triggers employees to enter the alarm stage. At this stage, employees experience heightened psychological distress, leading them to use impression management as coping strategies to manage the threat posed by negative supervisor gossip at the resistance stage. While impression management can swiftly enhance an individual’s public appearance through self-change (38, 41), the tactic demands a significant expenditure of an individual’s personal resources as they change themselves to promote a desirable public appearance and to maintain a certain consistency to avoid self-image contradictions (23). Over time, this process depletes psychological and cognitive resources of employees (24, 44, 45), leading them to experience emotional exhaustion at the exhaustion stage.

In sum, this study suggests that impression management is a mediating mechanism that explains the indirect effect of perceived negative supervisor gossip on employee emotional exhaustion. Therefore, this study proposes the following hypothesis:

*Hypothesis 3*: Employee impression management mediates the relationship between perceived negative supervisor gossip and emotional exhaustion.

### The moderating role of task interdependence

Task interdependence refers to the extent to which employees’ obligations, responsibilities, job-related tasks, and performance rely on other team members (47–50). This study adopts this definition to argue that task interdependence may moderate the effect of perceived negative supervisor gossip on impression management.

Supervisor gossip behaviors mainly consists of critical and condemnatory evaluations directed at specific employees (18, 27), which has strong individual pertinence. When employees perceive negative gossip behaviors from their supervisors, they may interpret it as an indication that their job-related performance or behavior has not met their supervisor’s expectations (15, 18). In cases where job responsibilities are highly individualized, perceived negative gossip is highly personal, leading employees to experience psychological stress and engage in impression management tactics to counteract potential reputational damage. However, when tasks and responsibilities are shared among team members, the personal relevance of the gossip is reduced, as employees may attribute the negative evaluation to the team as a whole rather than themselves individually, which would not induce as much psychological stress and impression management tactics as individual tasks.

Based on the task independence studies, this study suggests that employees with high task interdependence are better able to cope with perceived negative supervisor gossip than those with low task interdependence. Specifically, high task interdependence fosters a collective work identity, where employees perceive themselves and their team members as an integrated unit (25, 50). In this situation, employees are more likely to interpret the positive or negative evaluations as a collective honor or disgrace of the whole team, reducing the stress associated with negative supervisor gossip. Consequently, employees with high task interdependence may experience less stress from perceived negative supervisor gossip due to the mitigation of individual pertinence, so such employees may not engage in additional impression management behaviors.

Conversely, this study suggests that employees with low task interdependence may suffer more stress from perceived negative supervisor gossip, and exhibit more impression management tactics due to they view negative supervisor gossip as a direct personal critique. Specifically, employees with low task interdependence have autonomous job responsibilities, meaning that negative evaluations reflect solely on their individual performance (25, 48, 50). Therefore, employees may suffer higher stress from perceived negative supervisor gossip due to the increased individual pertinence, resulting in additional impression management behaviors.

In summary, this study proposes that task interdependence moderates the positive relationship between perceived negative supervisor gossip and impression management. We propose the following hypothesis:

*Hypothesis 4*: The relationship between perceived negative supervisor gossip and employee impression management is moderated by task interdependence, such that the direct effect is stronger when task interdependence is low.

Furthermore, this study proposed a moderated mediation model in which task interdependence moderates the positive effect of perceived negative supervisor gossip on emotional exhaustion through impression management. Specifically, high task interdependence means the negative supervisor gossip is mitigated by the individual pertinence, implying that the positive effect would be alleviated on impression management and even emotional exhaustion. Conversely, employees who experience low task interdependence would suffer stronger individual pertinence from negative supervisor gossip, meaning its positive effect would be strengthened on impression management and emotional exhaustion. Therefore, we propose the following hypothesis:

*Hypothesis 5*: The mediating effect of perceived negative supervisor gossip on emotional exhaustion through employee impression management is moderated by task interdependence, such that the mediating effect is stronger when task interdependence is low.

## Methods

### Participants and procedures

To decrease the common method biases Podsakoff et al. (51), the data were collected from Chinese employees working full-time spanning different job positions across multiple industries, including manufacturing, high-tech, finance, telecommunications, retail, and general services.

Building on the work of Griep et al. (52), the negative effects of work stress require a certain period to accumulate before significantly impacting emotional exhaustion. Previous studies have shown that the lagged effect of stressful events on emotional states typically peaks within 2 to 4 weeks (52). Based on these findings, this study implemented a one-month interval for data collection, as this period is crucial for capturing the key stages of stress accumulation. Accrodingly, after reaching cooperation with the HR of each company, the data were collected through a two-wave time-lagged design spanning 3 months during the second quarter of 2024, using online Chinese questionnaires.

Moreover, to increase respondent motivation, we established a lottery system whereby participants who completed all waves of the survey would have the chance to win a convenience store voucher. In Wave 1, we distributed 550 questionnaires and received 474 responses in first month, for a response rate of 86.18%. We also collected the last six digits of the respondents’ phone numbers as identification codes. One month later, we distributed Wave 2 questionnaires to the participants who completed questionnaires in Wave 1. For Wave 2, we received 406 responses, for a response rate of 85.65%. Thus, our final response rate was 73.82%.

The participant demographics were as follows: 253 females (62.3%) and 153 males (37.7%); 308 of the participants were 31–50 years old (75.9%), 94 of them were 18–30 years old (23.3%), and the rest were over 51 years old; the majority of them had a bachelor’s degree or higher (88.4%); 183 of the participants were general staff (45.1%); 203 of the participants had 11–20 years’ tenure (50.0%), 117 of them had 6–10 years’ tenure (28.8%), 59 of them had 0–5 years’ tenure (14.5%), and 27 of them had more than 20 years’ tenure (6.70%).

### Measures

All scales were developed in English. To ensure the translation accuracy, all items for each measurement were translated using the translation–back translation procedure (53) by four bilingual scholars in management.

#### Perceived negative supervisor gossip (Wave 1)

In Wave 1, a 12-item supervisor gossip scale (18) was used to capture the negative supervisor gossip behavior perceived by the employee. We also collected the positive supervisor gossip behavior perceived by the employee as a control variable. The scale contained six items each for positive (Cronbach’s *α* = 0.88) and negative supervisor gossip (Cronbach’s α = 0.89). A sample item for positive supervisor gossip was, “My supervisor discusses topics related to employees’ dedication and diligence at work.” A sample item for negative supervisor gossip was, “My supervisor discusses topics related to employees’ poor job performance.” These participant scores were captured using a 6-point Likert-type scale that ranged from 1 (“strongly disagree”) to 6 (“strongly agree”).

#### Task interdependence (Wave 1)

We captured task interdependence in Wave 1 by a 3-item scale developed by Liden et al. (25), with a Cronbach’s α of 0.87. A sample item was, “The way individual members perform their jobs has a significant impact upon others in the group.” The participant scores were captured using a 6-point Likert-type scale that ranged from 1 (“strongly disagree”) to 6 (“strongly agree”).

#### Impression management (Wave 2)

Impression management was measured by a 22-item impression management scale (42) in Wave 2. A sample item was, “Stay at work late so people will know you are hard working.” The participant scores were captured using a 6-point Likert-type scale that ranged from 1 (“strongly disagree”) to 6 (“strongly agree”). The Cronbach’s α of the impression management scale was.77.

#### Emotional exhaustion (Wave 2)

We measured emotional exhaustion with the 5-item Maslach burnout inventory (MBI-GS) developed by Maslach et al. (24) in Wave 2. The Cronbach’s α of MBI-GS was.92. A sample item was, “I feel used up at the end of the workday.” The participant scores were captured using a 7-point Likert-type scale that ranged from 0 (“never”) to 6 (“every day”).

#### Control variables (Wave 1)

We controlled for perceived positive supervisor gossip and the following demographic variables, which otherwise could have affected our hypothesized model: gender, age, educational level, position level, and tenure.

### Statistical methods

Several statistical tools were employed for data analysis. Specifically, SPSS 25.0 software was used to conduct descriptive statistics, reliability analysis, and correlation coefficient statistics of the sample. AMOS 21 software was utilized for model comparison to ensure that our hypothesized model was the best fit for the study. Additionally, PROCESS macro software was applied to test all research hypotheses.

## Results

### Descriptive statistics and correlation coefficient statistics

Table 1 provides a summary of the means, standard deviations (SD), bivariate correlations, and Cronbach’s alphas of all our study variables. Table 1 shows that perceived negative supervisor gossip was positively correlated with employee impression management (*r* = 0.28, *p* < 0.001) and emotional exhaustion (*r* = 0.10, *p* < 0.05). Impression management was positively correlated with emotional exhaustion (*r* = 0.41, *p* < 0.001). Therefore, the direct relationships of Hypotheses 1 and 2 were initially significant in terms of their correlation coefficient statistics.

**Table 1:** Variables’ means, standard deviations, reliabilities, and correlations (*N* = 406).

Variables	Mean	SD	1	2	3	4	5	6	7	8	9	10
1. Gender†	1.62	0.49										
2. Age†	2.86	0.68	−0.10									
3. Education†	5.14	0.68	−0.05	0.02								
4. Position†	1.96	1.03	−0.17^***^	0.36^***^	0.15^**^							
5. Organizational Tenure†	3.47	0.87	−0.04	0.74^***^	0.03	0.43^***^						
6. Perceived Positive Supervisor Gossip	4.52	0.91	−0.05	0.09	0.02	0.08	0.07	(0.88)				
7. Perceived Negative Supervisor Gossip	3.47	1.11	−0.08	0.19^***^	0.08	0.21^***^	0.22^***^	0.45^***^	(0.89)			
8. Impression Management	2.64	0.65	−0.22^***^	0.03	0.06	0.11^*^	0.04	0.19^***^	0.28^***^	(0.77)		
9. Emotional Exhaustion	2.75	1.03	−0.05	−0.07	0.04	−0.16^**^	−0.10^*^	0.04	0.10^*^	0.41^***^	(0.92)	
10. Task Interdependence	5.20	0.75	−0.08	0.08	0.00	0.11^*^	0.10^*^	0.38^***^	0.10^*^	−0.07	−0.06	(0.87)

Internal consistency (Cronbach’s alpha, α) is presented along the diagonal. ^†^Gender (1 = male, 2 = female); Age (1 = 18–25 years old, 2 = 26–30 years old, 3 = 31–40 years old, 4 = 41–50 years old, 5 = 50–60 years old, 6 = 60 years old above above); Education (1 = primary school or below, 2 = high school, 3 = (vocational) senior high school, 4 = junior college; 5 = bachelor’s degree, 6 = postgraduate or above); Position (1 = staff, 2 = junior manager, 3 = mid-level manager, 4 = senior manager); Organizational tenure (1 = 0–1 years, 2 = 1–5 years, 3 = 6–10 years, 4 = 11–20 years, 5 = 20 years above). *p < 0.05, **p < 0.01, ***p <. 001.

### Model analyses

Table 2 shows the results of our study’s model comparison. We conducted a model comparison test with parceling rules (54) in AMOS 21 software to test whether our hypothesized four-factor model had the best model fit with the collected data. Table 2 shows the results of the overall model analyses, which indicated the hypothetical model with four factors had a good fit with the data [*x^2^* (48) = 132.99; comparative fit index (CFI) = 0.97; incremental fit index (IFI) = 0.96; Tucker-Lewis index (TLI) = 0.97; root mean square error of approximation (RMSEA) = 0.07]. Table 2 also shows the results of the other four alternative-factors models. The goodness-of-fit statistical results indicate that the hypothetical model had a better model fit for our data collection.

**Table 2:** Results of the confirmatory factor analyses of the measures (N = 406).

Model	Factors	χ^2^	df	△χ^2^/df	CFI	IFI	TLI	RMSEA
Hypothetical Model	4 Factors	132.99	48		0.97	0.96	0.97	0.07
Model 1	3 Factors	416.06	51	94.36	0.85	0.81	0.85	0.13
Model 2	3 Factors	557.38	51	141.46	0.80	0.80	0.74	0.16
Model 3	2 Factors	1146.74	53	202.75	0.58	0.56	0.45	0.23
Model 4	1 Factor	1533.57	54	233.43	0.40	0.40	0.27	0.26

Bootstrap sample size = 50,000. Hypothetical Model (4 factors: perceived negative supervisor gossip, impression management, emotional exhaustion, and task interdependence). Model 1 (3 factors: perceived negative supervisor gossip, impression management and emotional exhaustion merged, and task interdependence). Model 2 (3 factors: perceived negative supervisor gossip and impression management merged, emotional exhaustion, and task interdependence). Model 3 (2 factors: perceived negative supervisor gossip, impression management and task interdependence merged, and emotional exhaustion). Model 4 (1 factor: all variables are merged into one factor).

### Hypotheses tests

**Figure 2:**
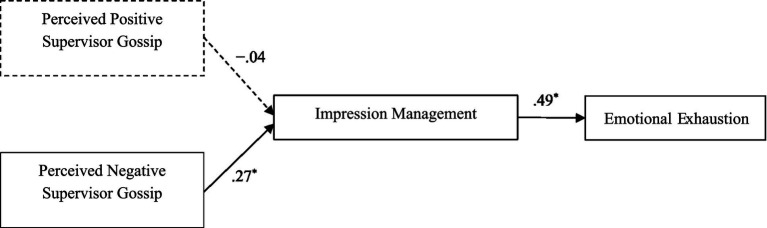
Hypothetical model results. *N* = 406. Standardized regression coefficients are reported. Bootstrap sample size = 50,000.

Structural equation modeling (SEM) software were used to test this study’s direct effect and mediation hypotheses. We controlling for the demographic variables and perceived positive supervisor gossip. This model tested the relationships among perceived negative supervisor gossip, impression management, and emotional exhaustion. Hypothesis 1 proposed that perceived negative supervisor gossip positively relates to employee impression management behaviors. Figure 2 shows our results, which indicate a strong positive relationship between perceived negative supervisor gossip and impression management (*β* = 0.27, *p* < 0.05). Hence, Hypothesis 1 was supported. Hypothesis 2 posited that employee impression management is positively correlated with emotional exhaustion. According to the results shown in Figure 2, the coefficient supported Hypothesis 2 (*β* = 0.49, *p* < 0.05).

We further used the Structural equation modeling (SEM) software and PROCESS macro software (55) to test the mediating effect of Hypothesis 3, which predicted that the relationship between perceived negative supervisor gossip and emotional exhaustion is mediated by impression management. Based on the results of SEM in Figure 2, this study further used the bootstrapping approach and Sobel test (56) in the PROCESS macro (55) to reconfirm the coefficient of the indirect effects of perceived negative supervisor gossip on emotional exhaustion through impression management. The results indicated that impression management mediated the relationship between perceived negative supervisor gossip and emotional exhaustion (*indirect effect* = 0.10, *p* < 0.001). Specifically, 50,000 bootstrapping approach tests indicated that the 95% confidence intervals (95% CIs) of the mediating effect was (0.05, 0.15). Therefore, Hypothesis 3 was supported.

**Figure 3:**
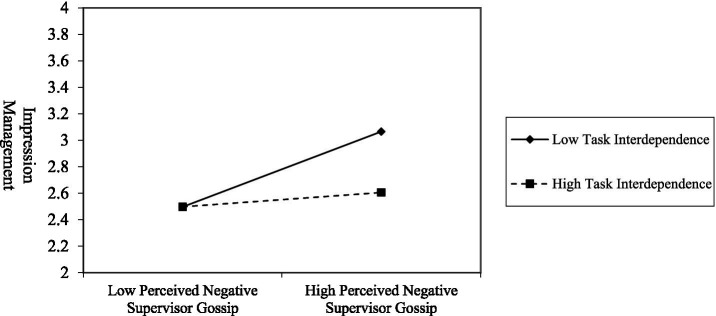
Moderating effect of task interdependence on the relationship between perceived negative supervisor gossip and impression management.

Hypothesis 4 suggested that task interdependence moderates the direct effect between perceived negative supervisor gossip and impression management. This study tested a simple moderating effect using PROCESS macro software (55). Table 3 shows that the interaction was statistically significant of task interdependence and perceived negative supervisor gossip (*β* = −0.14, 95% CI = [−0.21, −0.07]). We further examined the conditional effects by dividing task interdependence into three groups to represent the moderator at low (−1 SD), mean, and high (+1 SD) levels. Table 3 shows that the conditional effect was significant only at the moderator’s low (*β* = 0.26, 95% CI = [0.17, 0.35]) and mean levels (*β* = 0.16, 95% CI = [0.10, 0.22]). Figure 3 shows the moderating effects of task interdependence on the relationship between perceived negative supervisor gossip and impression management. Therefore, Hypothesis 4 was supported.

**Table 3:** Regression results for moderation (*N* = 406).

Task interdependence	b	SE	Boot LL 95%CI	Boot UL 95% CI
Values for task interdependence in the simple moderated effect for impression management				
−1 SD	0.26	0.05	0.17	0.35
Mean	0.16	0.04	0.10	0.22
+1 SD	0.05	0.04	−0.03	0.13
Simple moderation index	−0.14	0.04	−0.21	−0.07
Task interdependence moderated mediation results for emotional exhaustion				
−1 SD	0.18	0.04	0.11	0.26
Mean	0.11	0.03	0.06	0.16
+1 SD	0.04	0.03	−0.02	0.09
Moderated mediation index	−0.10	0.03	−0.16	−0.04

Unstandardized regression coefficients are reported. Bootstrap sample size = 50,000. LL, lower limit; CI, confidence interval; UL, upper limit.

Hypothesis 5 further predicted that task interdependence moderates the indirect effect of perceived negative supervisor gossip on emotional exhaustion through impression management. Hypothesis 5 was also tested by the PROCESS macro software (55). Table 3 indicates that the index was significant of the conditional indirect effects of moderated mediation (*β* = −0.10, 95% CI = [−0.16, −0.04]). Similarly, the moderated mediation effect was significant at the low (*β* = 0.18, 95% CI = [0.11, 0.26]) and mean levels (*β* = −0.10, 95% CI = [−0.16, −0.04]). Hence, Hypothesis 5 was supported.

## Discussion

This study integrated insights from general adaptation syndrome theory to examine the influence of perceived negative supervisor gossip on employee impression management tactics and subsequent emotional exhaustion. As predicted, our empirical results show that employee’s perceptions of negative supervisor gossip are positively correlated with impression management, which positively affects employee emotional exhaustion. This result indicates the mediating role of impression management as an underlying process between perceived negative supervisor gossip and emotional exhaustion. That is, supervisors who frequently engage in negative employee gossip may cause their employees to deplete more of their personal resources as they engage in more impression management behaviors, resulting in employee emotional exhaustion. Furthermore, the findings provide insights for organizational behavior practices by highlighting task interdependence as an intervention to mitigate the impact of perceived negative supervisor gossip.

### Theoretical implications

This study makes four significant theoretical contributions, offering deeper insights into the effects of perceived negative supervisor gossip on employee emotional exhaustion.

First, occupational health is a prominent topic in the field of organizational behavior. The prior research has mainly investigated the effect of workplace gossip on employees’ occupational health outcomes, such as mental health (15) or subjective well-being (14). The relationship between workplace gossip and emotional exhaustion has been largely overlooked. This study addresses this gap by exploring how perceived negative supervisor gossip influences employee emotional exhaustion. Our findings indicate that negative gossip from supervisors acts as a stressor that leading to emotional exhaustion through impression management. By highlighting this relationship, our study advances the occupational health literature by demonstrating that workplace gossip extends beyond work behaviors and psychological effects, directly contributing to employee emotional exhaustion. Moreover, this study focuses on negative supervisor gossip due to the unique power dynamics. Compared to peer gossip, supervisor gossip carries more authority and evaluative weight, making it more likely to increase employees’ psychological stress. This finding highlights the significance of gossip sources, indicating that supervisor gossip, as an authoritative stressor, may have a stronger psychological impact than peer gossip.

Second, previous workplace gossip studies have predominantly focused on gossip among employees rather than supervisor gossip about employees (10, 13, 27). Drawing inspiration from (18), this study frames workplace gossip within the supervisor–subordinate information exchange process and examines employees’ reactions to perceived negative gossip from their supervisors. Previous studies have mainly focused on examining the impact of workplace gossip on employee attitude and psychological states, such as cynicism (11, 13), leader–member exchange (18), and psychological capital (15). However, less research has emphasized employees’ coping behaviors after perceiving negative supervisor gossip. Hence, this study reveals that employees adopt impression management as a coping strategy to mitigate the stress caused by perceived negative supervisor gossip. This finding extends the literature by demonstrating that negative gossip from supervisors not only undermines employee psychological well-being but also prompts behavioral responses to prevent the reputational damage caused by negative supervisor gossip.

Third, this study not only aligns with the research showing that impression management can lead to employee emotional exhaustion (23, 35, 40), but it also examines the role of impression management as a mediator between perceived negative supervisor gossip and emotional exhaustion. Specifically, this study drawing upon general adaptation syndrome theory (19) to argue that employees perceive negative supervisor gossip as a workplace stressor that triggers them entering the alarm stage. In response, during the resistance stage, employees may engage in impression management tactics to counteract the psychological distress of the potential reputational damage. Finally, with the personal resources depletion of impression management, employee may experience emotional exhaustion at exhaustion stage of stress. By integrating general adaptation syndrome theory with impression management, our findings offer a novel perspective on how employees attempt to mitigate the stress from negative supervisor gossip and the unintended negative consequences of these coping mechanisms.

Finally, this study explores the moderating effect of task interdependence on the relationship among perceived negative supervisor gossip, impression management, and emotional exhaustion. This study demonstrates that employee with high task interdependence can mitigates the negative effects of perceived negative supervisor gossip, reducing employees’ reliance on impression management and consequently alleviating emotional exhaustion. Therefore, this finding not only extend the literature of negative supervisor gossip, impression management, emotional exhaustion, and task interdependence, but also offers valuable insights for the implementation of task interdependence in managerial practices.

## Practical implications

This study also has several implications for organizational behavior and managerial practices. First, our findings suggest that negative gossip from supervisors is linked to employee stress and emotional exhaustion. As various studies have noted, workplace gossip cannot be entirely eliminated (10, 13, 27), managers should focus on promoting efforts to minimize instances of supervisors’ negatively gossiping about their employees as much as possible. For instance, organizations can establish formal communication guidelines and implement anti-negative-gossip provisions into organizational ethics policies to explicitly discourage negative gossiping behaviors by supervisors (32). Moreover, since positive gossip can have a positive impact on employees’ desirable attitudes and behaviors (15, 18), supervisors can consider engaging in positive rather than negative employee gossip. In addition, when employees experience negative supervisor gossip, the human resources department can offer counseling services or act as a mediator to resolve conflicts between the supervisor and the employee (14).

Second, this study our findings highlight the role of impression management as a mediator in the relationship between perceived negative supervisor gossip and emotional exhaustion. Since impression management depletes employees’ personal resources (26, 41), managers should be trained to recognize signs of excessive impression management. Moreover, regular employees’ mental well-being assessments can help managers spot early signs of employee exhaustion. When needed, they can offer support like adjusting workloads, providing mental health resources, or encouraging open and authentic communication to ease the stress on employees.

Finally, our study underscores the protective role of task interdependence in mitigating the detrimental effects of perceived negative supervisor gossip. Employees in highly interdependent work environments experience lower stress from perceived negative supervisor gossip, engage in fewer impression management behaviors, and consequently suffer less emotional exhaustion. Therefore, organizations should actively foster collaborative work environments by promoting interaction and teamwork (47, 48, 50). Additionally, the human resource department can emphasize the benefits and significance of teamwork and enhance collaboration among employees within the organization through job redesign strategies (49, 50).

## Limitations and future directions

This study also has several limitations. First, our research focuses solely on perceived negative supervisor gossip, without considering the potential impact of negative gossip from colleagues. Future studies could control peer workplace gossip to test the influence of supervisor gossip on employee outcomes.

Second, we measured supervisor gossip at the subordinate level by capturing the subordinate’s perceptions of supervisor gossip. However, supervisor gossip may also have cross-level impacts. Therefore, future research must establish a cross-level research framework to investigate the influence of supervisor gossip on employees.

Third, to avoid common method biases (51), we collected our data with a time-lagged design. However, although the research has found the relationship between perceived negative supervisor gossip, impression management behavior, and employee emotional exhaustion (23, 26, 35, 38, 40), this study cannot draw a causal conclusion because we did not collect data for all variables in every wave, which means we cannot consider variables perceived negative supervisor gossip and impression management behavior from each wave as control variables. Hence, future studies should further examine whether a causal relationship persists between perceived negative supervisor gossip, impression management, and emotional exhaustion.

Forth, our study also relies on survey-based data, which is inherently retrospective and may be subject to measurement biases. While surveys provide valuable insights, they cannot fully establish causal relationships. Moreover, according to the curve properties of the general adaptation syndrome (GAS) theory, the relationship and potential mechanism between perceived negative supervisor gossip and employee emotional exhaustion may be a nonlinear curve relationship. However, our cross-sectional design could not identify the gradual or cumulative process that perceived negative supervisor gossip leads to emotional exhaustion. Therefore, future study could conduct experimental research, which could enhance the robustness. Moreover, experimental research could allow for more direct causal inferences and a more controlled investigation into the curve dynamics of workplace gossip on emotional exhaustion.

Finally, this research tested only the one alternative underlying mechanism of impression management and the one boundary condition of task interdependence. More alternative underlying processes and boundary conditions need to be explored to elucidate the relationship between perceived negative supervisor gossip and emotional exhaustion. For instance, employees may engage in retaliatory behavior (57) by resorting to actions aimed at undermining others as a coping strategy to alleviate the stress caused by perceived negative supervisor gossip. Moreover, trust among colleagues may serve as a potential moderator since higher levels of mutual trust tend to enhance the evaluation of information’s authenticity in informal communications involving team members (58). Moreover, this study was conducted in the Chinese cultural context, which emphasizes collectivism and encourages collaboration (59). In such a cultural context, the moderating effect of task interdependence is well-established. Future study could further explore this model in individualistic cultures, where individual independence is highly valued, to identify whether the moderated effect of task interdependence would become notably stronger. Furthermore, if the relationship between stress and emotional exhaustion is not strictly linear, certain moderating variables, such as personal resilience or social support, may influence the transition from the resistance stage to exhaustion. Therefore, future research could explore these moderating effects in greater depth, potentially using experimental designs to better capture the curve dynamic of this process.

## Conclusion

Gossip is common in the professional environment, and all individuals at all levels participate. The research has mainly focused on gossip at the colleague level. However, this study investigated the impact of supervisor gossip on employees from the supervisor–subordinate perspective. The results suggest that organizational behavior and managerial practices should shed light on how to effectively manage negative supervisor gossip and how to intervene effectively in mitigating its negative impact on employees.

## Data Availability

The raw data supporting the conclusions of this article will be made available by the authors, without undue reservation.

## References

[1] Cheng H Fan Y Lau H. An integrative review on job burnout among teachers in China: implications for human resource management. Int J Hum Resour Manag. (2023) 34:529–61. doi: 10.1080/09585192.2022.2078991

[2] Lee JS Akhtar S. Job burnout among nurses in Hong Kong: implications for human resource practices and interventions. Asia Pac J Hum Resour. (2007) 45:63–84. doi: 10.1177/1038411107073604

[3] Kim H Lee SY. Supervisory communication, burnout, and turnover intention among social workers in health care settings. Soc Work Health Care. (2009) 48:364–85. doi: 10.1080/00981380802598499, PMID: 19396707

[4] Ninaus K Diehl S Terlutter R. Employee perceptions of information and communication technologies in work life, perceived burnout, job satisfaction and the role of work-family balance. J Bus Res. (2021) 136:652–66. doi: 10.1016/j.jbusres.2021.08.007

[5] Rajesh JI Suganthi L. The satisfaction of teachers with their supervisors’ interpersonal communication skills in relation to job burn-out and growth satisfaction in southern India. Manag Educ. (2013) 27:128–37. doi: 10.1177/0892020613498521

[6] Schaufeli WB Peeters MC. Job stress and burnout among correctional officers: a literature review. Int J Stress Manag. (2000) 7:19–48. doi: 10.1023/A:1009514731657

[7] Ter Hoeven CL van Zoonen W Fonner KL. The practical paradox of technology: the influence of communication technology use on employee burnout and engagement. Commun Monogr. (2016) 83:239–63. doi: 10.1080/03637751.2015.1133920, PMID: 27226694 PMC4867864

[8] Thomas CH Lankau MJ. Preventing burnout: the effects of LMX and mentoring on socialization, role stress, and burnout. Human Resour Manag. (2009) 48:417–32. doi: 10.1002/hrm.20288

[9] Chang K Kuo C-C. Can subordinates benefit from Manager’s gossip? Eur Manag J. (2021) 39:497–507. doi: 10.1016/j.emj.2020.09.009

[10] Foster EK. Research on gossip: taxonomy, methods, and future directions. Rev Gen Psychol. (2004) 8:78–99. doi: 10.1037/1089-2680.8.2.78

[11] Kuo C-C Chang K Quinton S Lu C-Y Lee I. Gossip in the workplace and the implications for HR management: a study of gossip and its relationship to employee cynicism. Int J Hum Resour Manag. (2015) 26:2288–307. doi: 10.1080/09585192.2014.985329

[12] Naeem M Weng Q Ali A Hameed Z. An eye for an eye: does subordinates’ negative workplace gossip lead to supervisor abuse? Pers Rev. (2020) 49:284–302. doi: 10.1108/PR-05-2018-0174, PMID: 35579975

[13] Kuo C-C Chang K Kuo T-K Chen S. Workplace gossip and employee cynicism: the moderating role of dispositional envy. Chinese J Psychol. (2020) 62:5. doi: 10.6129/CJP.202009_62(4).0005

[14] Cheng B Peng Y Zhou X Shaalan A Tourky M Dong Y. Negative workplace gossip and targets’ subjective well-being: a moderated mediation model. Int J Hum Resour Manag. (2023) 34:1757–81. doi: 10.1080/09585192.2022.2029931

[15] Cheng S Kuo C-C Chen H-C Lin M-C Kuo V. Effects of workplace gossip on employee mental health: a moderated mediation model of psychological capital and developmental job experience. Front Public Health. (2022) 10:881. doi: 10.3389/fpubh.2022.791902, PMID: 35493358 PMC9041444

[16] Tanner G Otto K. Superior–subordinate communication during organizational change: under which conditions does high-quality communication become important? Int J Hum Resour Manag. (2016) 27:2183–201. doi: 10.1080/09585192.2015.1090470

[17] Vanderstukken A Schreurs B Germeys F Van den Broeck A Proost K. Should supervisors communicate goals or visions? The moderating role of subordinates' psychological distance. J Appl Soc Psychol. (2019) 49:671–83. doi: 10.1111/jasp.12626

[18] Kuo C-C Wu C-Y Lin C-W. Supervisor workplace gossip and its impact on employees. J Manag Psychol. (2018) 33:93–105. doi: 10.1108/JMP-04-2017-0159

[19] Selye H. The general adaptation syndrome and the diseases of adaptation. J Clin Endocrinol. (1946) 6:117–230. doi: 10.1210/jcem-6-2-117, PMID: 21025115

[20] Fletcher D Hanton S Mellalieu SD. An organizational stress review: Conceptual and theoretical issues in competitive sport. New York, NY: Nova Science Publishers (2008).

[21] Sarp N Yarpuzlu AA Önder ÖR. A comparative study of organizational stress in three hospitals in Ankara. Turkey *Stress Health*. (2005) 21:193–7. doi: 10.1002/smi.1055

[22] Gardner WL Martinko MJ. Impression management in organizations. J Manag. (1988) 14:321–38. doi: 10.1177/014920638801400210

[23] Vohs KD Baumeister RF Ciarocco NJ. Self-regulation and self-presentation: regulatory resource depletion impairs impression management and effortful self-presentation depletes regulatory resources. J Pers Soc Psychol. (2005) 88:632–57. doi: 10.1037/0022-3514.88.4.632, PMID: 15796665

[24] Maslach C Jackson SE Leiter M. P. (1997). Maslach Burnout Inventory: Third edition. In Zalaquett C. P. Wood R. J. (Eds.), Evaluating stress: A book of resources. Scarecrow Education, 191–218.

[25] Liden RC Wayne SJ Bradway LK. Task interdependence as a moderator of the relation between group control and performance. Hum Relat. (1997) 50:169–81. doi: 10.1023/A:1016921920501

[26] Klotz AC He W Yam KC Bolino MC Wei W Houston L III. Good actors but bad apples: deviant consequences of daily impression management at work. J Appl Psychol. (2018) 103:1145–54. doi: 10.1037/apl0000335, PMID: 29939036

[27] Kurland NB Pelled LH. Passing the word: toward a model of gossip and power in the workplace. Acad Manag Rev. (2000) 25:428–38. doi: 10.2307/259023

[28] Zhu Q Martinescu E Beersma B Wei F. The double-edged sword of negative supervisor gossip: when and why negative supervisor gossip promotes versus inhibits feedback seeking behavior among gossip targets. Hum Relat. (2023) 77:864–86. doi: 10.1177/00187267231165885, PMID: 40103853

[29] Gkorezis P Petridou E Xanthiakos P. Leader positive humor and organizational cynicism: LMX as a mediator. Leadership Organ Dev J. (2014) 35:305–15. doi: 10.1108/LODJ-07-2012-0086

[30] Kark R Van Dijk D. Motivation to lead, motivation to follow: the role of the self-regulatory focus in leadership processes. Acad Manag Rev. (2007) 32:500–28. doi: 10.5465/amr.2007.24351846

[31] Xie J Huang Q Wang H Shen M. Coping with negative workplace gossip: the joint roles of self-monitoring and impression management tactics. Personal Individ Differ. (2019) 151:109482. doi: 10.1016/j.paid.2019.06.025

[32] Liu X-Y Kwan HK Zhang X. Introverts maintain creativity: a resource depletion model of negative workplace gossip. Asia Pac J Manag. (2020) 37:325–44. doi: 10.1007/s10490-018-9595-7

[33] Furnham A Capon M. Social skills and self-monitoring processes. Personal Individ Differ. (1983) 4:171–8. doi: 10.1016/0191-8869(83)90017-X

[34] Schlenker BR Pontari BA. The strategic control of information: impression management and self-presentation in daily life In: Tesser A Felson RB Suls JM, editors. Psychological perspectives on self and identity: American Psychological Association (2000). 199–232. doi: 10.1037/10357-008

[35] Bolino MC. Citizenship and impression management: good soldier or good actors? In: Angle HL Porter LW Allen RW, editors. Organizational influence processes. London: Routledge (2016). 82–98.

[36] Jones EE Pittman TS. Toward a general theory of strategic self-presentation. Psychol Perspect Self. (1982) 1:231–62.

[37] Duck S. (1991). Friends, for life: The psychology of personal relationships (2nd ed.). London: Harvester Wheatsheaf.

[38] Leary MR Kowalski RM. Impression management: a literature review and two-component model. Psychol Bull. (1990) 107:34–47. doi: 10.1037/0033-2909.107.1.34

[39] Berman JZ Levine EE Barasch A Small DA. The Braggart's dilemma: on the social rewards and penalties of advertising prosocial behavior. J Mark Res. (2015) 52:90–104. doi: 10.1509/jmr.14.0002

[40] Peng AC Gao R Wang B. Linking servant leadership to follower emotional exhaustion through impression management. J Organ Behav. (2023) 44:643–59. doi: 10.1002/job.2682

[41] Bolino M Long D Turnley W. Impression management in organizations: critical questions, answers, and areas for future research. Annu Rev Organ Psych Organ Behav. (2016) 3:377–406. doi: 10.1146/annurev-orgpsych-041015-062337

[42] Bolino MC Turnley WH. Measuring impression management in organizations: a scale development based on the Jones and Pittman taxonomy. Organ Res Methods. (1999) 2:187–206. doi: 10.1177/109442819922005

[43] Bande B Jaramillo F Fernández-Ferrín P Varela JA. Salesperson coping with work-family conflict: the joint effects of ingratiation and self-promotion. J Bus Res. (2019) 95:143–55. doi: 10.1016/j.jbusres.2018.10.015

[44] Fitriastuti T. (2019). Job burnout as a consequence of impression management and Ocb: moderating role of leader–member exchange. Academy of Management Global Proceedings.

[45] Alarcon GM. A meta-analysis of burnout with job demands, resources, and attitudes. J Vocat Behav. (2011) 79:549–62. doi: 10.1016/j.jvb.2011.03.007

[46] Demerouti E Bakker AB Nachreiner F Schaufeli WB. The job demands-resources model of burnout. J Appl Psychol. (2001) 86:499–512. doi: 10.1037/0021-9010.86.3.499, PMID: 11419809

[47] Bachrach DG Powell BC Collins BJ Richey RG. Effects of task interdependence on the relationship between helping behavior and group performance. J Appl Psychol. (2006) 91:1396–405. doi: 10.1037/0021-9010.91.6.1396, PMID: 17100493

[48] Kiggundu MN. Task interdependence and the theory of job design. Acad Manag Rev. (1981) 6:499–508. doi: 10.2307/257385

[49] Liden RC Erdogan B Wayne SJ Sparrowe RT. Leader-member exchange, differentiation, and task interdependence: implications for individual and group performance. J Organ Behav. (2006) 27:723–46. doi: 10.1002/job.409

[50] Van der Vegt GS Janssen O. Joint impact of interdependence and group diversity on innovation. J Manag. (2003) 29:729–51. doi: 10.1016/S0149-2063_03_00033-3

[51] Podsakoff PM MacKenzie SB Lee J-Y Podsakoff NP. Common method biases in behavioral research: a critical review of the literature and recommended remedies. J Appl Psychol. (2003) 88:879–903. doi: 10.1037/0021-9010.88.5.879, PMID: 14516251

[52] Griep Y Vranjes I Kraak JM Dudda L Li Y. Start small, not random: why does justifying your time-lag matter? Span J Psychol. (2021) 24:e45. doi: 10.1017/SJP.2021.42, PMID: 34511144

[53] Brislin RW. Back-translation for cross-cultural research. J Cross-Cult Psychol. (1970) 1:185–216. doi: 10.1177/135910457000100301

[54] Marsh HW Lüdtke O Nagengast B Morin AJ Von Davier M. Why item parcels are (almost) never appropriate: two wrongs do not make a right—camouflaging misspecification with item parcels in CFA models. Psychol Methods. (2013) 18:257–84. doi: 10.1037/a0032773, PMID: 23834417

[55] Hayes A. F. (2017). Introduction to mediation, moderation, and conditional process analysis: A regression-based approach. New York, Guilford publications.

[56] Preacher K. J. Leonardelli G. J. (2001). Calculation for theSobel test: An interactive calculation tool for mediation tests [Computersoftware]. Available at: https://www.unc.edu/*preacher/sobel/sobel.htm (Accessed July 14, 2005).

[57] Skarlicki DP Folger R. (2004). Broadening our understanding of organizational retaliatory behavior. In Griffin R. W. O’Leary-Kelly A. M. (Eds.), The dark side of organizational behavior, San Francisco: Jossey Bass. 373–402.

[58] Mellinger GD. Interpersonal trust as a factor in communication. J Abnorm Soc Psychol. (1956) 52:304–9. doi: 10.1037/h0048100, PMID: 13318834

[59] Yau-Fai Ho D Chiu CY. (1994). Component ideas of individualism, collectivism, and social organization: an application in the study of Chinese culture. Cross Cultural Research And Methodology Series, 18, 137–137.

